# ‘We don’t know nearly enough’: an online survey exploring perspectives of specialists who support children with brain-based visual impairments

**DOI:** 10.3389/fnhum.2024.1510812

**Published:** 2025-01-29

**Authors:** Josephine Sabrina Jakubowski, Eloise May, Rebecca Findlay, Nicola McDowell, Samantha K. Simkin, Lisa M. Hamm

**Affiliations:** ^1^Department of Medicine, Queen’s University, Kingston, ON, Canada; ^2^School of Nursing, University of Auckland, Auckland, New Zealand; ^3^School of Optometry and Vision Science, University of Auckland, Auckland, New Zealand; ^4^Blind and Low Vision Education Network New Zealand (BLENNZ), Auckland, New Zealand; ^5^Institute of Education, Massey University, Auckland, New Zealand

**Keywords:** brain based visual impairment, CVI, cerebral visual impairment, parent perspectives, survey, transdisciplinary care

## Abstract

**Introduction:**

Children with brain-based visual impairments (some of whom have a diagnosis of Cerebral Visual Impairment, or ‘CVI’) represent a growing and underserved population within vision services. These children often have more complex needs than those with ocular visual impairments and benefit from specialist support from multiple disciplines. This study aimed to understand the perspectives of these specialists in terms of their goals, views on collaboration, and understanding of the term ‘CVI’.

**Methods:**

We invited a range of specialists who work with children with brain-based visual impairments, including educators, rehabilitation staff, clinicians, and family members, to complete an online survey between April 2023 and April 2024.

**Results:**

The analysis included 94 respondents: 51 educators, 30 rehabilitation staff, 7 clinicians, and 6 family members. Respondents shared common goals of connecting with the child (87/94, 93%) and fostering their learning and development (82/94, 93%). However, respondents also noted some specific and divergent goals, which can be at odds with each other. Professional staff frequently identified family members as the most valuable source of information about their child’s vision (36/88, 41%), though family members expressed feeling under-valued. Transdisciplinary clinics were highlighted as a helpful model to provide quality child-centered care. Of the 73 professional staff who reported being familiar with the term ‘CVI’ (73/88, 83%), most (61/73, 84%) thought it was underdiagnosed, but respondents had different perspectives on what a diagnosis meant. Only 73% of professionals familiar with CVI reported receiving formal training about it.

**Discussion:**

The varied goals and different perspectives on CVI create challenges to providing cohesive support for children with brain-based visual impairments. Increasing the availability of complementary formal training across disciplines and adopting transdisciplinary models of care are promising approaches to improve the quality of services.

## 1 Introduction

When low vision originates within the eye, it typically limits the visual information accessible to a child, manifesting as reduced visual acuity, diminished contrast sensitivity, or restricted visual fields. When visual challenges originate in the brain, these foundational elements of vision may be limited, but impairments can also alter visual attention, how visual information is interpreted and how it is integrated with other sensory inputs (Lueck et al., 2019; Philip and Dutton, 2014). These challenges are harder to measure and articulate, so they can lead to misunderstandings, and necessitate different approaches to low vision support (Brown et al., 2024; McDowell, 2023; McDowell and Budd, 2018). Although strategies and frameworks are being developed to understand what children with brain-based visual impairments see, and how to best support these children (McDowell, 2023; Pilling et al., 2023; Zatta and Willems, 2024), there are not yet standardized guidelines (Pilling et al., 2023).

Terminology is also an area of ongoing debate (Chang et al., 2024; Costa, 2024; Kran et al., 2019; Pilling et al., 2023; Sakki et al., 2018), with ‘cortical’ and ‘cerebral’ commonly used, both shortened to ‘CVI’. Here, we use the term ‘brain-based visual impairments’ broadly to include children who struggle to process visual information but may not have a formal diagnosis, and ‘CVI’ more specifically to refer to a diagnosed condition. Recent collaborative initiatives to standardize terminology (Chang et al., 2024) and develop assessment protocols (Zatta and Willems, 2024) are essential and timely, given the growing impact of brain-based impairments on childhood low vision and blindness. In 2000, CVI was the cause of 50% of severe visual impairment and blindness in UK children, rising to 61% by 2015 – a trend echoed in other high-income countries (Teoh et al., 2023).

Brain-based visual impairments rarely occur in isolation; children may have had congenital or acquired brain injuries, neurological malformations, and/or genetic conditions, resulting in co-occurring motor, communication and cognitive challenges (Philip and Dutton, 2014). Whether the manifestations of CVI are co-occurring with, critical components of, or causative factors for other neurodevelopmental conditions is another area of ongoing debate (Chokron and Dutton, 2023; Chokron et al., 2020). Given the co-occurring challenges, children living with brain-based visual impairments benefit from the support of a range of specialists, including ophthalmologists, pediatricians, rehabilitation therapists, educators, community support networks and their families. Each specialist requires a comprehensive understanding of each child’s strengths and challenges, as well as how they use their vision day to day to reach their goals, however approaches to understanding what a child sees can be very different (Bennett et al., 2019; Silveira, 2019).

In this study we sought to explore perspectives of specialists who support children with brain-based visual impairments. We aimed to understand (1) the goals specialists have when trying to understand what children with brain-based visual impairments see, (2) perceptions about the flow of information between specialists, and (3) how specialists understand the term ‘CVI’.

## 2 Methods

### 2.1 General

The cross-sectional survey was conducted online using Qualtrics (Qualtrics, 2022; Utah, USA), with recruitment (by email invitation) between April 2023 and April 2024. Qualtrics allows for IP address checking to prevent reporting of duplicate responses. The study is reported using relevant items of CROSS (Sharma et al., 2021) and STROBE (Von Elm et al., 2009) guidelines.

### 2.2 Pre-testing

The survey was piloted by multiple stakeholders with different roles, including caregivers of children with brain-based visual impairment, educators, rehabilitation and clinical staff, and researchers. Feedback was incorporated into the survey iteratively. Key changes included a broadening of scope (resulting in findings presented across two manuscripts) and modifying terminology to reflect international differences.

### 2.3 Participants

We used purposive, convenience sampling by emailing a project summary and survey link to leadership at relevant organizations within authors’ networks, requesting that it be shared further within their organizations as appropriate. These organizations included parent groups for children with visual impairments, educators of visually impaired children, vision rehabilitation service providers, as well as clinical collaborators. This indirect recruitment strategy precluded reporting of the total number of participants invited to share their perspectives.

When potential participants accessed the survey, they were provided with information about the study, inclusion criteria, and associated definitions. To meet inclusion criteria, participants must have supported at least one child (0–18 years) with suspected or known brain-based visual impairment in a professional or personal capacity. Respondents self-identified as meeting these criteria and provided consent before accessing survey questions. No identifying information was collected, but specific role, country and free text answers were removed from data to preclude identification.

### 2.4 Data collection

The questions emerged from stakeholder input. A full list of questions for this report is included as a Supplementary material.

### 2.5 Defining terms

In the information provided before the survey, participants were informed that we used ‘brain-based’ visual impairments broadly to encompass a variety of terms, and specifically noted that a child did not require a formal diagnosis of CVI for a specialist to participate. Within the survey, we specified that we use ‘brain-based’ to include a known or suspected neurological origin, ‘visual’ to include a wide range of visual abilities (not limited to visual acuity), and ‘impairment’ to refer to challenges impacting the child when both eyes are open. Respondents were asked to describe the children they interacted with (including whether they had a CVI diagnosis) and shared their perspectives on the meaning and implications of the term CVI.

### 2.6 Predictors/potential confounder/effect modifiers

We used the respondent’s self-identified role to contextualize responses, grouping specific roles into the broader categories of family, education, rehabilitation, and clinical staff.

### 2.7 Analysis

#### 2.7.1 Quantitative

We report descriptive statistics for all quantitative questions. Single-answer and multi-select questions are presented by response frequency. Five-point Likert scales were used to quantify agreement with statements, ranging from ‘strongly agree’ (coded as 2), to ‘strongly disagree’ (coded as −2), with neutral responses coded as zero. These codes were used to calculate the mean and standard deviation for relevant questions.

#### 2.7.2 Qualitative

For the free-text questions, similar responses were grouped together, and themes were generated iteratively. Key themes were highlighted with selected quotes.

## 3 Results

### 3.1 Respondent characteristics

**Figure 1:**
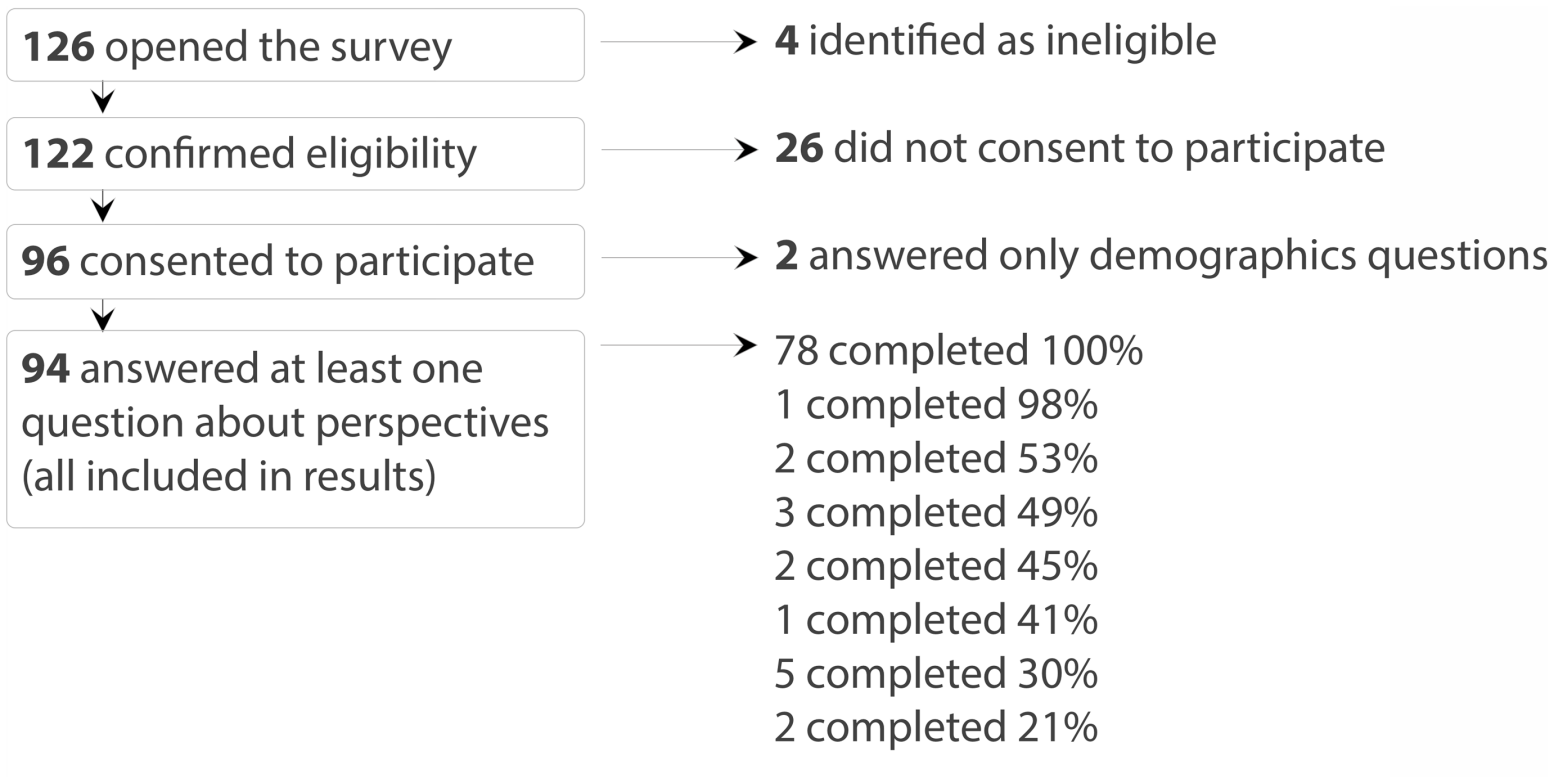
Participation among respondents commencing the survey.

Of the 126 individuals who opened the online survey, 122 indicated they were eligible to participate. Ninety-six respondents consented to take part in the survey, and 94 completed questions beyond the demographics section and were included in the analysis. Of these 94 participants, 78 (83%) completed the entire survey (Figure 1).

Table 1 summarizes basic demographic information of the 94 respondents. Most were based in Aotearoa, New Zealand (78/94, 83%), with the remainder in the United States, Canada, and Australia. Most respondents came from an education background (51/94, 54%), including specialized teachers for vision, teaching assistants, and classroom teachers, with specialized teachers for vision the most selected role (43/94, 46%). Those who identified as rehabilitation staff (30/94, 32%) mainly included physiotherapists and occupational therapists. Only seven respondents reported a clinical role (including optometrists, ophthalmologists and pediatricians), and six were family members of children with brain-based visual impairment.

**Table 1:** Demographic information of 94 respondents included in analysis.

Country	*n*	%
Aotearoa, New Zealand	78	83%
United States of America	7	7%
Canada	6	6%
Australia	3	3%
Role		
Education	51	54%
Rehabilitation	30	32%
Clinical	7	7%
Family	6	6%
Setting		
School	64	68%
Home	16	17%
Clinic	12	13%
Other	2	2%

Of the 87 respondents who answered questions about severity, only 10 said they mostly supported children with mild visual impairments (10/87, 11%), the rest mostly supported children with moderate (49/87, 56%) or severe (27/87, 31%) visual processing challenges. Consistent with the more severe visual challenges, most respondents reported interacting with children who diagnosed with CVI (85/87, 98%), and co-occurring motor (76/87, 87%), cognitive (76/87, 87%), and communication (72/87, 83%) challenges.

Families reported daily interactions with children, while rehabilitation professionals typically reported weekly interactions, and educators mostly reported monthly interactions (consistent with a higher representation of specialized teachers for visually impaired children than classroom teachers/teaching assistants). In contrast, clinicians reported annual interactions. The duration of each individual interaction corresponded with its frequency: family members spent full or half days, educators and rehabilitation staff spent several hours, and clinical professionals reported spending about an hour per interaction.

### 3.2 Goals

Respondents were asked why it was important to understand what children with brain-based visual impairments see. They were provided five options and asked to select all that applied, followed by a request to select which goal was most important to them. Respondents were also provided a free text option to expand on their goals.

Most respondents selected multiple answers, with the two most common goals being “to better understand, or connect with a child” (87/94, 93%) and “to help the child develop an aspect of vision or wider development” (82/94, 87%). When asked to choose which goal was most important among selected options, educators and rehabilitation staff most frequently prioritized supporting learning and development (Educators: 25/51, 49%, Rehabilitation staff: 12/30, 40%), clinicians most frequently aimed to set expectations for a child’s future visual capacity (4/7, 57%), and family members most frequently reported wanting to understand what their child sees to better connect with them (4/6, 67%).

Free-text responses revealed more specific and divergent goals, with clear alignment to professional role. For example, some classroom teachers emphasized the need to support access to educational curriculum. Specialized teachers for vision focused on supporting families by providing accurate information about what the child sees. Occupational therapists noted the importance for functional planning. Speech-language therapists reported wanting to understand vision to provide suitable communication options. One respondent articulately observed that these more divergent priorities can be at odds with each other, and highlighted the importance of collaboration to overcome tension resulting from differing goals:

*“It is really important for speech language therapists to collaborate more with low vision specialists, as our priorities are often at odds when we are implementing [communication] systems for children with complex communication needs and CVI. We need to work together to find compromises.” – Rehabilitation staff*

Respondents shared common goals of connecting with a child and supporting their development. However, free text responses revealed more divergent practical goals, which can create tension. Where goals are at odds, collaboration was highlighted as a crucial tool. Working together can help refocus specialists on shared goals and navigate compromises to achieve the best outcomes for children.

### 3.3 Collaboration

Most of our respondents (71/92, 77%) felt like they already worked as part of a collaborative interdisciplinary team, and the value of these networks was frequently highlighted in their comments. For instance, one respondent emphasized the positive impact on families:

*“…we are working truly in a transdisciplinary model which is very exciting - as it strongly includes the whānau [family, broadly defined] and keeps the child at the centre.” – Education staff*

Despite this high level of perceived collaboration among our respondents, on average, our respondents strongly agreed that they would like to collaborate with other specialists more (Mean 1.6 ± StDev 0.7), and respondents were, on average, neutral to the statement *“I do not think I have the time/resources to collaborate with other specialists more”* (Mean 0.0 ± StDev 1.3). Although overall motivation to collaborate more was stronger than reported time/resource constraints, these practical constraints were still cited as barriers in the free text, particularly in more rural or isolated areas:

*“In the national team I am part of there is wonderful transdisciplinary sharing and collaboration. In my regional hospital role, I feel that there is further need to develop this cross disciplinary working model and while there is interest to do this time and resource gets in the way, as well as developing new ways of working. I think this is especially true for small centres where people have to be more generalist in their roles (like optometrists and ophthalmologists not being specifically paediatric, and paediatricians not being developmental etc).” – Clinical staff*

Respondents were asked about information flow, including sources and recipients of information. They were provided a list of specialists and asked to select all that they received information about a child’s vision from, followed by a request to select which specialist provided the most useful information about what a child sees. Respondents were then asked to select all the specialists they provided information to about what a child sees. There was also a free text option to expand on information sharing.

On average, respondents reported receiving information from eight different specialists (StDev±5), most commonly including the family (78/94, 83%), and the child’s ophthalmologist (71/94, 76%). Respondents reported providing information to seven different specialists (StDev±5), mostly commonly the family (79/94, 84%), and the child’s classroom teacher (69/94, 73%). Family members were unique in that they reported providing more information than they received about their child’s vision.

Across all respondents, family members were highlighted most frequently as the most valuable source of information (37/94, 39%). By specialist group, educators (25/51, 49%) and clinicians (4/7, 57%) most frequently reported family as the most valuable source of information about what a child with brain-based visual impairment sees.

The groups reported to provide the most valuable information about what a child with brain-based visual impairment see (family, low vision specialists and specialist teachers for visually impaired students), revealed in free text that they were uncertain if their contributions were valued. Specialist teachers for vision noted spending considerable time writing reports on how children with brain-based impairments use their vision daily yet were provided little feedback about the value this information held for other specialists. Likewise, families reported lack of feedback about the value of their insights, one family member articulated their hope in this domain:

*“I would like to be more involved and be listened to more and be treated as a person who provides valuable information about my child.” – Family member*

Taken together, most of our respondents reported feeling part of a collaborative interdisciplinary team, and they wanted to further invest in these teams because the value for the family was clear. However, setting up this way of working was highlighted as a challenge, especially outside urban where resources are stretched and there is less domain specific expertise. Importantly, those who are providing most of the information to the network of specialists aren’t getting the feedback that it is valued, which has the potential to erode these valuable collaborative networks.

### 3.4 Understanding of CVI

Seventy-eight respondents (78/94, 83%) reported they were familiar with the term ‘CVI’. Among those who were, most (69/78, 90%) believed a CVI diagnosis was important, mostly for accessing services (40/69, 58%) but also to help with understanding/identity (29/69, 42%). There was broad consensus that CVI is currently under-diagnosed (64/78, 82%). However, opinions on what constitutes a diagnosis of CVI varied. Respondents were divided on whether CVI should be considered a collection of ‘symptoms’/‘manifestations’ (31/78, 40%), or whether a diagnosis should reflect something atypical about the health of the visual system (31/78, 40%), the remaining 20% offered other interpretations of what CVI. Twenty-two percent of respondents thought that evidence or history of damage in the brain was required for a CVI diagnosis (17/78, 22%) while only 12% believed that reduced visual acuity was required for a diagnosis (9/78, 12%). Clinical professionals, who are responsible for diagnosing CVI, were more likely to view the diagnosis as indicative of the visual system’s health and to require reduced visual acuity and evidence of neurological deviation. These differing perspectives on CVI likely contribute to tension among specialists.

*“CVI is not 'diagnosed' in most cases…it would be helpful if ophthalmologists have more up to date knowledge around CVI and provide it in reports if possible.” – Education staff*

Of the 73 professional staff who were familiar with the term CVI, 53 (73%) reported having formal training on CVI, this equates to 60% of all professional staff included in our analysis. Respondents detailed informal ways they learned about CVI, including websites (notably the CVI Scotland website), social media and organic learning from other professionals and interactions with children living with brain-based visual impairments. Respondents employed within the education sector appeared to have the more opportunities for updated professional development on CVI, which was valued by those receiving it. However, this highlighted a knowledge gap between clinical and educational staff.

*“It often seems that others, even vision professionals, know less about CVI than we do, and we don't know nearly enough!” – Education staff*

Overall, respondents tended to agree that a diagnosis of CVI was important for appropriate service provision (69/78, 90%), and it was underdiagnosed (64/78, 82%). Appropriately increasing the number of diagnoses is difficult when specialists have different ideas of what should constitute a CVI diagnosis, and education about CVI is largely informal, and often self-directed.

## 4 Discussion

### 4.1 Key results summary

Promoting a child’s development and finding meaningful ways to connect with them were common overarching goals of the specialists surveyed; people caring for children with brain-based visual impairments want them to thrive. Many of our respondents felt they were part of collaborative teams, and specifically highlighted the power of transdisciplinary teams for providing quality services for children with brain-based visual impairments. However, several barriers to quality care were identified.

The logistics of professionals from different disciplines working together can be limited by time and resource constraints. In settings where this infrastructure exists, moving from multi-disciplinary to trans-disciplinary requires strong interpersonal rapport. Our data highlights some factors that could be barrier to these collaborative relationships. First, specialists have different specific goals, which can be at odds with one another. Second, those providing information about how a child uses their vision day to day do not always feel that their contributions are valued. Finally, specialists have different ideas about what a diagnosis of CVI means. These interpersonal factors can erode the collaborative relationships that are so key to transdisciplinary work.

The responses highlighted the need for enhanced formal training about CVI. If core elements of this training are consistent across disciplines, it would help align perspectives. Opportunities for formal teaching about the goals, responsibilities, and values of other disciplines, and how they can work together, would lay the groundwork for integrated support, where all members of multi-disciplinary teams are valued for the expertise they bring.

### 4.2 Connection to wider literature

The need for more formal training about brain-based visual impairments is aligned with other research. Studies that quantify training time in professional programs suggest training about CVI is limited. Castleberry et al. (2021) quantified CVI education in Ophthalmology and Optometry courses and found that only about half of programs (48%, 23/48) offer formal didactic CVI lectures, most only including 1–2 h. They found clinical instruction on CVI was sporadic and trainees typically first encounter CVI in pediatric eye care clinics and low vision clinics. The limited training about CVI has been reflected in generally poor knowledge in survey studies (Maitreya et al., 2018; Stanimirov et al., 2021). Similarly, Harpster et al. (2022) found a significant gap in formal CVI education for occupational therapists and teachers of students with low vision managing CVI.

Although there is a long way to go, awareness of CVI among diagnosing clinicians has improved over time. In a 2010 survey, parents reporting needing to fight to get a diagnosis (Jackel et al., 2010), while in a 2019 follow-up survey, parents reported the diagnostic process was easier and more timely. However, general knowledge was still lacking. In 2019, parents reported being given very little information about what the diagnosis means (Jackel, 2019). Similarly, interviews with parents in 2023 still indicated professional staff had poor knowledge about CVI (Oliver et al., 2023). Survey data from 2021 showed that parents who receive information at time of diagnosis report better rapport with care providers and feel more empowered to advocate for their child (McDowell, 2021), demonstrating the importance of clear information.

Increasing the hours of training within discipline is important for families, but developing a complementary curriculum across disciplines would help take the onus off the family to integrate information from different specialists. It would also help specialists understand the goals, responsibilities and values of specialists in other roles, promoting realistic expectations and better collaboration. The term ‘transdisciplinary’ was raised by several respondents in our survey to refer to meaningful collaborative networks centered around the child. While ‘multidisciplinary’ refers to a group that includes members from a variety of disciplines and ‘interdisciplinary’ refers to when the concepts from each discipline are integrated into a new whole, ‘transdisciplinary’ refers to collaborative networks that transcend disciplinary boundaries (Choi and Pak, 2006).

This transdisciplinary collaboration has been an important concept in early intervention for a long time (reviewd by King et al., 2009). There are known costs to implement transdisciplinary services in terms of managerial and team resources, however the benefits for children, families, and staff are considerable (King et al., 2009). The value of collaboration between health and education specifically is also recognized in the wider literature, with tangible steps for implementation. For example Burgess et al. (2015, using school immunisation as a case study) highlight that meaningfully integrating approaches requires substantial time, high levels of trust, and a willingness to share turf. The concept of transdisciplinary work is also highlighted in the CVI literature, though not always by name. Interviews with parents and children with CVI highlighted a central challenge for parents is integrating information between the specialists, and learning how to use this information to advocate for their child (Goodenough et al., 2021). Putting the onus on the service providers to communicate integrated information would reduce this pressure on families who already face substantial challenges.

### 4.3 Limitations

A strength of this study was the diverse range of specialists invited to participate, including family members, educators, rehabilitation, and clinical staff. Our recruitment was most successful within a blind and low vision education organization in Aotearoa, New Zealand. This organization generally works with children who have visual impairments meeting criteria for support, and they prioritize holistic service and collaboration between disciplines. Consequently, our results reflect an education-centric perspective, and we have limited input from clinicians and families. Our results also reflect the experience of working with children who have more severe impairments, and those who have some experience working as part of an inter- or trans- disciplinary team. This group may not fully represent the broader specialist community. Although our findings may underestimate the challenges faced by specialists outside such well-integrated networks, our data have the benefit of providing insights into the potential of transdisciplinary collaboration.

### 4.4 Future directions

Summarizing how different specialists respond to the same questions about brain-based visual impairments has highlighted some shared goals, as well as potential tensions between disciplines. This could be further elucidated by specifically sampling groups of specialists to compare perspectives quantitatively. As work is done to develop guidelines, and professional curriculum across disciplines (Pilling et al., 2023), continued research is key to knit together cohesive and complementary information and approaches.

## 5 Conclusion

Appropriately diagnosing, understanding, and supporting children with brain-based visual impairments is complex and requires a wide range of specialists to work together. These specialists tend to have different specific goals, responsibilities, and understanding of what CVI is, and these differences can lead to tensions between disciplines. Although clinical staff reported the value of family member’s and educators’ perspectives, families and educators do not always feel that their input is valued, and can question whether clinicians are taking their concerns about the children they support seriously.

Families and specialist teachers for vision feel the burden of educating themselves and others about brain-based visual impairments, despite often feeling uncertain, and not having decision-making power related to access to services. To alleviate this burden and utilize all specialists’ desire to see children develop and thrive, clear guidelines and more cohesive education about brain-based visual impairments is needed. Improving knowledge about the condition and about the roles, responsibilities and value of other specialists, fostering cross-disciplinary care networks, and providing a context where interactions can become transdisciplinary, are potential pathways to higher quality care for children with brain-based visual impairments.

## Data Availability

The raw data supporting the conclusions of this article will be made available by the authors, without undue reservation.
