# Supplementary material for: ‘We don’t know nearly enough’: an online survey exploring perspectives of specialists who support children with brain-based visual impairments
Source: Front Hum Neurosci. 2025 Jan 29;18:1510812. doi: 10.3389/fnhum.2024.1510812 (PMC11877649; doi:10.3389/fnhum.2024.1510812)
Supplement: Supplementary file 1 [file Data_Sheet_1.DOCX]

Survey questions

# Respondent characteristics

This section is about your context, and your role with children who have brain-based visual impairment.

What type of role best describes the way you support impacted children?

Role Options:

- Whānau | family member or caregiver
- Personal support worker
- General Practitioner / family doctor
- Paediatrician
- Developmental paediatrician
- Neurologist
- Psychologist
- Ophthalmologist
- Optometrist
- Orthoptist
- Nurse
- Social worker or related
- Physiotherapist
- Occupational therapist
- Low-vision specialist
- Speech language pathologist / therapist
- Audiologist
- Developmental orientation and mobility specialist
- Resource teacher for vision
- Early intervention specialist
- Teaching / educational assistant
- Classroom teacher
- Principal
- Service coordinator
- Researcher

If none of these roles quite fit, please add details below FREE TEXT

In which country do you currently reside? (pre-populated drop down list)

Where do you typically interact with children who have brain-based visual impairment?
*(select all that apply)*

*Options: Home, School,* Community centre, Clinic, Lab, Other *(free text)*

If more than one selected

Where is the most frequent location you interact with children with brain-based visual impairment?
*(select one)*

## Interactions with children who have brain-based visual impairments

(wording different for family and other specialists – professional staff wording provided)

Take a moment to consider the children with brain-based visual impairment who you support (as part of your role as a/an [role piped in])

Reminders of definition during survey: 
 1) We use 'brain-based' to include a known or suspected neurological origin

2) We use 'visual' to include a wide range of visual abilities (not limited to visual acuity)
 3) We use 'impairment' to refer to challenges impacting the child when both eyes are open

Which diagnostic categories do these children fall into?
 *(select all that apply)*

Options:

- Diagnosis of CVI ('cerebral' or 'cortical' visual impairment)
- Diagnosis of bilateral amblyopia (as a result of delayed treatment of early eye conditions such as cataract, or profound bilateral refractive correction)
- Diagnosis of a condition or history which puts the child at-risk of CVI (e.g. cerebral palsy, prematurity, seizures, brain injury, etc)
- Diagnosis of a neuro-developmental condition which can have visual processing symptoms (e.g. autism)
- Visual processing challenges not associated with any of the above diagnoses

Feel free to add any notes about diagnostic categories here. *(Note that we ask about perspectives on definitions of 'CVI' at the end of this survey)* FREE TEXT

What is the most common severity of**visual challenges** in the children you see with brain-based visual impairment?

Options:

- Severe (mostly visually unresponsive)
- Moderate (reliably visually responsive, but ability lower than typical)
- Mild (reliably visually responsive, ability typical in some ways, but can be lower than typical in others)

Do the children you support with brain-based visual impairment usually have **additional (non-visual) challenges?**
 *(select all that apply)*

*Options:*

- General motor challenges
- Cognitive challenges
- Communication challenges
- None of the above

Which **age groups** do these children fall into?
*(select all that apply)*

*Options:* 0-5 years, 6-12 years, 13-16 years, 17-18 years

How many **different** children with brain-based visual impairment do you interact with in your role, in a typical year?
Options: 1 child, 2-5 children, 6-20 children, 21-50 children, >50 children

Which frequency best describes your interactions with **an individual child** with brain-based visual impairment, in a typically year?

Options: Everyday, Once a week, Once a month, Once a year

Which best describes the length of a typical **single interaction with an individual child** with brain-based visual impairment?

Options: 15 minutes, 30 minutes, 1 hour, Half day, Full day

# Goals

In your role as a/n [role piped in]), why is it important for you to know what a child with brain-based visual impairment sees?
*(select all that apply and/or write in your own goals)*

Goals options:

- To better understand, or connect with a child
- To connect a child to the right support services and funding
- To provide insight about expectations for a child's visual abilities in the future
- To monitor development or rehabilitation
- To help the child develop an aspect of vision or wider development
- Other (free text)

If more than one selected:

Among these goals, which would you say is the most important to you?
(select one)

Add free text to describe your goals if not captured above

# Interactions between specialists

Children with brain-based visual impairment can interact with many different specialists. In this section, consider how you (in your role as a/an [role piped in]) share information with other specialists.

## Information flow

Who do you **receive** information about a child's visual abilities from?
*(select all that apply)*

Role list repeated

If more than one answer

Of the information you receive, which type of specialist provides the most useful information to you?

Role list repeated

Who do you **provide** information about a child's visual abilities to?
 *(select all that apply)*

Role list repeated

## Interdisciplinary Teams

In some contexts, multi-disciplinary assessment centers exist to help collaboration between specialists who have different perspectives (e.g. maybe clinical and allied health).
Do you feel like you have access to, or are part of, a multi-disciplinary team?

Options: Yes, No

To what extent do you agree with the following statement: I would like to collaborate with other specialists more.

Options: Strongly agree, Somewhat agree, Neither agree nor disagree, Somewhat disagree, Strongly disagree

To what extent to you agree with the following statement: I don't think I have the time/resources to collaborate with other specialists more.

Options: Strongly agree, Somewhat agree, Neither agree nor disagree, Somewhat disagree, Strongly disagree

If you have any thoughts about value of information shared with you, or how you feel the information you share with others is valued, please write them below

# Understanding CVI

The final block of questions if more theoretical, we are interested in your personal familiarity and perspectives with certain terms and ideas. You can skip this section if you like, but any insights you want to share would be really valuable.

Are you familiar with the term 'cerebral visual impairment' (sometimes 'cortical visual impairment' or CVI)?

Options: Yes, No

If yes:

Which best describes your thoughts about CVI?

Options:

- It is a term to identify a collection of symptoms
- It is a term to identify an abnormality in the visual system
- Other (free text)
- Not sure

Do you think that CVI is

Options: Over-diagnosed, Under-diagnosed, Generally diagnosed appropriately, Not sure

Do you think that visual acuity needs to be impaired for a child to have a diagnosis of CVI?

Options: Yes, No, Unsure

Do you think there needs to be verifiable damage, or clear history consistent with damage, to the brain for a child to have a diagnosis of CVI?

Options: Yes, No, Unsure

Do you think a diagnosis of CVI matters?

Options:

- Yes - mostly for children and whānau | families to promote understanding or identity
- Yes - mostly to access needed support for vision
- No - I don't really think a diagnosis matters
- Other (free text)

Where have you gotten your information about CVI from?
Options: Formal training, Informal information sharing (facebook, etc), Websites, Other (free text)

If more than one

Where have you gotten the most information about CVI from?
